# Pre-existingmental health disorders affect pregnancy and neonatal outcomes: a retrospectivecohort study

**DOI:** 10.1186/s12884-020-03094-5

**Published:** 2020-07-25

**Authors:** Kotryna Sūdžiūtė, Greta Murauskienė, Kristina Jarienė, Algirdas Jaras, Meilė Minkauskienė, Virginija Adomaitienė, Irena Nedzelskienė

**Affiliations:** 1 Faculty of medicine, Clinic of Psychiatry, Lithuanian University of Health Sciences, Medical Academy, Eivenių street 2, LT-50161 Kaunas, Lithuania; 2 Faculty of medicine, Clinic of Obstetrics and Gynecology, Lithuanian University of Health Sciences, Medical academy, Eivenių street 2, LT-50161 Kaunas, Lithuania; 3 Faculty of Odontology, Clinic of Dental and Oral Pathology, Lithuanian University of Health Sciences, Medical Academy, Eivenių Street 2, LT-50161 Kaunas, Lithuania

**Keywords:** pregnancy, mental disorders, antenatal care, delivery outcomes, neonatal outcomes

## Abstract

**Background:**

This was a hospital registry-based retrospective age-matched cohort study that aimed to compare pregnancy and neonatal outcomes of women with pre-existing mental disorders with those of mentally healthy women.

**Methods:**

A matched cohort retrospective study was carried out in the Department of Obstetrics and Gynecology, Hospital of Lithuanian University of Health Sciences Kauno Klinikos, a tertiary health care institution. Medical records of pregnant women who gave birth from 2006 to 2015 were used. The study group was comprised of 131 pregnant women with mental disorders matched to 228 mentally healthy controls. The primary outcomes assessed were antenatal care characteristics; secondary outcomes were neonatal complications.

**Results:**

Pregnant women with pre-existing mental health disorders were significantly more likely to have low education, be unmarried and unemployed, have a disability that led to lower working capacity, smoke more frequently, have chronic concomitant diseases, attend fewer antenatal visits, gain less weight, be hospitalized during pregnancy, spend more time in hospital during the postpartum period, and were less likely to breastfeed their newborns. The newborns of women with pre-existing mental disorders were small for gestational age (SGA) more often than those of healthy controls (12.9% vs. 7.6%, *p* < 0.05). No difference was found comparing the methods of delivery.

**Conclusions:**

Women with pre-existing mental health disorders had a worse course of pregnancy. Mental illness increased the risk to deliver a SGA newborn (RR 2.055, 95% CI 1.081–3.908).

## Background

Mental disorders are among the most common conditions affecting women of reproductive age. The World Health Organization recognizes that 10%−16% of pregnant women and 13%−20% of postpartum women worldwide experience mental disorders, and most of these women suffer from depression [1].

Untreated perinatal mental disorders have a significant negative impact on both maternal and fetal health [2]. Because of the changes in women’s neurochemistry caused by identical peptides and proteins synthesized by the brain and the placenta during pregnancy (brain-derived neurotrophic factor, oxytocin, vascular endothelial growth factor, cortisol, matrix metalloproteinase), women with untreated mental disorders (anxiety disorder, post-traumatic stress disorder, schizophrenia, depressive disorders) are at an increased risk of obstetric complications such as preterm birth and delivery of low-birth-weight and small-for-gestational-age (SGA) newborns [3]. In addition, there are multiple other factors that increase the probability for adverse delivery outcomes, including genetic predisposition, maternal stress, sociodemographic disadvantage, poor nutrition, addiction to psychoactive substances, and poor antenatal care (ANC) attendance [4–6].

Other studies suggest that women with mental illness have a higher rate of overall complications in their pregnancy. However, the findings on the risk to deliver SGA newborns are not consistent [7–12].

The aim of our study was to analyze sociodemographic, pregnancy and delivery outcomes of women with pre-existing mental disorders and to compare them with the outcomes of mentally healthy women as well as to determine whether women with pre-existing mental disorders had worse perinatal outcomes.

## Methods

A hospital registry-based retrospective matched cohort study, involving women with pre-existing mental disorders that gave birth in the Department of Obstetrics and Gynecology, Lithuanian University of Health Sciences Kauno Klinikos, from January 1, 2006, to December 31, 2015, was carried out. The study was approved by the Lithuanian Bioethics Committee (No. BEC-MG-173); the permission to collect medical records from the hospital was obtained as well.

The study used birth registry records that included basic information such as age, previous pregnancies and deliveries, and clinical diagnoses. Women with mental disorders were selected based on the International Classification of Diseases Codes (ICD-10), and medical records were retrieved from the medical archive.

The diagnoses of mental disorders (according to the ICD-10) in the study group were as follows: schizophrenia; schizotypical and delusional disorders (F20–F29); mood (affective) disorders (F30 − F39); neurotic stress related and somatoform disorders (F40–F48); mild mental retardation (F70); behavioral disorders due to use of psychoactive substances (F10 − F19); and other (Table 1). It was found that 32.1% (*n* = 41) of the women in the study group were using psychotropic medication during their pregnancy, namely antipsychotics (*n* = 16, 12.2%), antidepressants (*n* = 05, 3.8%), benzodiazepines (*n* = 07, 5.38%), and psychotropic polytherapy (*n* = 13, 9.9%). The majority of women who had the diagnosis of behavioral disorder due to the use of psychoactive substances used heroin or amphetamines (*n* = 11, 91.6%).

**Table 1:** The distribution of women by mental disorders in the study group

Mental disorders (diagnostic categories)	Study group *n* = 131
Schizotypical and delusional disorders	40 (30.5)
Mood (affective) disorders	41 (31.3)
Neurotic stress-related and somatoform disorders	18 (13.7)
Mild mental retardation	17 (13)
Behavioral disorder due to use of psychoactive substances	12 (9.2)
Other	3 (2.3)

Values are number (percentage)

**Fig. 1:**
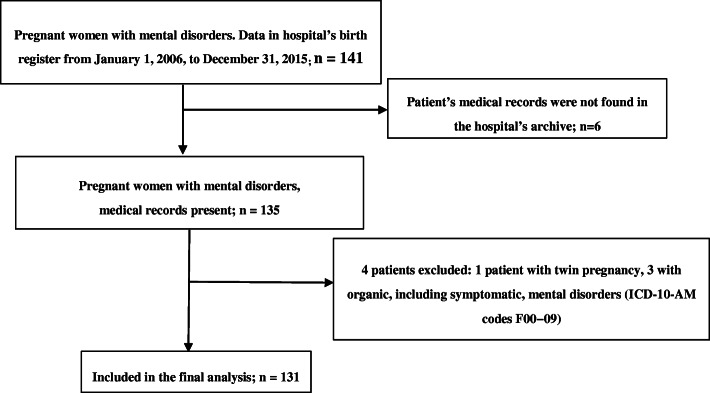
Flowchart of study participants, according to the hospital’s birth registry data

The total number of women who gave birth at the hospital during the period of January 1, 2006, to December 31, 2015, was 34,638. The final study group was comprised of 131 pregnant women with mental disorders. Patients with organic including symptomatic, mental disorders (ICD-0-AM codes F00 − 09) and twin pregnancies were excluded (Fig. 1).

Each participant from the study group was matched with two mentally healthy controls with single pregnancies, who were matched by age, year of delivery, and number of previous pregnancies and deliveries. A total of 262 control women were selected, but the final control group consisted of 228 pregnant women as the medical records of 34 women were missing from the hospital’s archive.

Data regarding women’s sociodemographic status, concomitant diseases, pregnancy complications and course, process of delivery and available data on newborns were used in the analysis.

Statistical analysis was performed with the IBM SPSS Statistics v21 program using independent samples, Kolmogorov–Smirnov, chi-square and Mann − Whitney tests. Multiple logistic regression models were constructed to determine the effect of mental disorders on various dependent variables. The degree of error was set below 5% as the threshold of statistical significance (*p* < 0.05).

## Results

Of the 403 medical records of pregnant women, 359 fulfilled the inclusion criteria. The mean maternal age at delivery did not differ significantly between the study group and healthy controls (30 ± 6.4 vs. 30 ± 5.9 years). The number of previous deliveries and pregnancies also did not differ significantly (Table 2).

**Table 2:** Maternal characteristics

	Characteristics	Study group *n* = 131	Healthy controls *n* = 228	*p*
Sociodemographic characteristics	Age, mean (SD), years	30 (6.4)	30 (5.9)	ns
	Previous deliveries, mean (SD), n	1 (1.3)	1 (1.1)	ns
	Previous pregnancies, mean (SD), n	2 (2.6)	2 (1.5)	ns
	Primary education	41 (31.2)	18 (8)	< 0.001
	Higher education	32 (24.8)	132 (58)	< 0.001
	Employed	40 (31)	167 (73.6)	< 0.001
	Married	60 (45.7)	170 (74.4)	< 0.001
	Single	71 (54.2)	58 (25.4)	< 0.001
	Concomitant somatic diseases	92 (69.9)	83 (36.6)	< 0.001
	Smoking	45 (34.1)	19 (8.4)	< 0.001
Pregnancy characteristics	Unknown LMP	16 (12.2)	6 (2.6)	< 0.05
	Did not seek ANC	25 (19)	3 (1.3)	< 0.05
	ANC visits, mean (SD), n	6 (4.1)	8 (3.1)	< 0.05
	Weight gain < 10 kg	68 (51.9)	36 (15.9)	< 0.05
	UTIs	5 (3.8)	1 (0.4)	ns
	Pyelonephritis	4 (3.1)	1 (0.4)	ns
	Hypertension	4 (3.1)	12 (5.3)	ns
	Hypertension with eclampsia	5 (3.8)	6 (2.6)	ns
	GDM	7 (5.3)	10 (4.4)	ns
	Hospital admission	73 (55.7)	69 (30.3)	< 0.001
	Vaginal delivery	104 (79.4)	175 (76.8)	ns
	Elective caesarean section	11 (8.4)	26 (11.4)	ns
	Emergency caesarean section	16 (12.2)	27 (11.8)	ns
	Epidural analgesia	28 (21.4)	63 (27.6)	ns

Values are number (percentage) unless otherwise stated. *LMP *last menstrual period; *ANC *antenatal care; *UTIs *urinary tract infections; *GDM* gestational diabetes mellitus

Women with mental disorders were more likely to have primary education (31.2% vs. 8.0%; *p* < 0.001), to be single (54.2% vs. 25.4%, *p* < 0.001), to have lower working capacity (33.8% had lower working capacity because of their mental illness), and less likely to be employed (31% vs. 73.6%; *p* < 0.001) than their healthy counterparts. In addition, women with mental disorders more often had concomitant somatic diseases (69.9% vs. 36.6%; *p* < 0.001) and smoked during pregnancy (34.1% vs. 8.4%; *p* < 0.001) (Table 2).

The women of the study group more often did not seek ANC (19% vs. 1.3%; *p* < 0.05), had fewer ANC visits (6 ± 4.1 vs. 8 ± 3.1; *p* < 0.05) (the mean number of ANC visits for multiparas and primiparas approved by the Lithuanian Ministry of Health is 7 and 10, respectively), and had higher rates of hospital admissions during pregnancy (55.7% vs. 30.3%; *p* < 0.001). However, the incidence rate of UTIs, pyelonephritis, hypertension, hypertension with eclampsia and gestational diabetes mellitus did not differ between the groups (Table 2).

There was no difference in the methods of delivery. Moreover, mentally ill women and healthy controls did not differ significantly regarding the rates of vaginal delivery, elective and emergency CSs, and epidural analgesia. Women with mental disorders spent significantly more days in the hospital in the postpartum period (*p* < 0.05): the average was 6 days after elective CS, 8 days after emergency CS, and 5 after vaginal delivery compared with the control group with the corresponding averages of 5, 7, and 4 days.

The newborns of the mentally ill women were small for gestational age more often (12.9% vs. 7.6%; *p* < 0.05). Other adverse outcomes such as prematurity, stillbirths, major malformations, low birth weight, and the Apgar scores lower than 7 after 5 min were more common in the study group, but without significant difference. The women with mental disorders were less likely to breastfeed their newborns (71.3% vs. 94.3%; *p* < 0.001) (Table 3).

**Table 3:** Newborns’ characteristics

Characteristics	Study group *n* = 131	Healthy controls *n* = 228	*p*
Prematurity	21 (16.4)	26 (11.5)	n s
Stillbirth	5 (3.8)	4 (1.8)	n s
Major malformations	2 (0.6)	0 (0)	n s
SGA newborn	17 (12.9)	17 (7.6)	< 0.05
LBW	19 (15.1)	22 (9.8)	n s
Apgar scores after 5 min < 7	6 (4.8)	10 (4.5)	n s
Breastfed	93 (71.3)	215 (94.3)	< 0.001

Values are number (percentage). *SGA *small for gestational age; *LBW *low birth weight

After adjusting the model of logistic regression, the data revealed mental disorders to be a significant predictor of lower education, unemployment, being unmarried, having chronic concomitant diseases, smoking, hospitalization during pregnancy, weight gain of < 10 kg, and lower ANC attendance. As regards neonatal outcomes, mental disorders were a predictor of having an SGA newborn and the choice to not breastfeed (Table 4).

**Table 4:** Variables predicted by mental diseases

Variables	*p*	Relative risk	95% CI
Lower education	< 0.001	4.051	2.508 − 6.543
Unemployment	< 0.001	6.37	3.963 − 10.238
Unmarried	< 0.001	3.371	2.135 − 5.322
Chronic concomitant diseases	< 0.001	3.976	2.38 − 6.643
Smoking	< 0.001	3.976	2.428 − 6.511
Hospital admission	< 0.001	2.9	1.857 − 4.529
Weight gain of < 10 kg	< 0.001	2.93	1.877 − 4.575
Lower ANC attendance	< 0.001	1.918	1.233 − 2.985
SGA newborns	< 0.05	2.055	1.081–3.908
Not breastfed	< 0.001	6.758	3.44–13.274

*ANC *antenatal care; *SGA *small for gestational age

To avoid possible bias and the impact of concomitant diseases as well as psychotropic medication used, adjusted statistical analysis was performed. The results remained the same with some slight differences (Table 5).

**Table 5:** Adjusted analysis of variables predicted by mental diseases

Variables	*P* value		Relative risk	95% CI
Lower education	< 0.001		8.467	3.911 − 18.328
Unemployment	< 0.001		9.941	5.017 − 19.697
Unmarried	< 0.001		3.49	1.917 − 6.354
Smoking	< 0.001		5.156	2.666 − 9.972
Hospital admission	< 0.001		1.812	1 − 3.284
Weight gain of < 10 kg	< 0.001		3.139	1.734 − 5.684
Lower ANC attendance	< 0.001		2.874	1.482 − 5.570
SGA newborns	< 0.05		2.482	1.126 − 5.471
Not breastfed	< 0.001		6.312	2.766 − 14.408

*ANC *antenatal care; *SGA *small for gestational age

Five (3.8%) women from the study group were transferred to the psychiatric department postpartum due to exacerbation of mental disorder. One woman had a behavioral disorder due to the use of psychoactive substances (heroin); other had a diagnosis of schizophrenia and experienced a psychotic episode after delivery. The remaining three women were originally hospitalized to the psychiatric department, and after delivery, they were transferred back due to a lack of progress in the treatment of their mental disorder (hebephrenic schizophrenia, reoccurring depressive disorder, and bipolar affective disorder).

## Discussion

The morbidity rates due to mental disorders are high in Lithuania, and in 2014 they were 2.5 times higher than the European average [13]. However, this study, analyzing pregnancy and neonatal outcomes of women with pre-existing mental disorders, was the first to be conducted in Lithuania.

Our data suggest that pregnant women with mental disorders were more likely to have lower social status and were more susceptible to tobacco use during pregnancy. The relative risk of low education and unemployment was 4 and 6 times higher, respectively, in women with mental disorders than healthy controls. The reason behind such behavior remains unclear as it is very hard to determine whether it is due to the mental illness itself or a social shift associated with it. These women did not receive adequate ANC during their pregnancy as the study group had low ANC attendance rates and engaged in dangerous activities, such as usage of psychoactive substances and smoking. Multiple studies have reported the correlation between the mental health of a pregnant woman and risky behavior during pregnancy, for example lower ANC attendance [14, 15]. In our study, there were more smokers among women with mental disorders than healthy controls (34.1% vs. 8.4%), and women with mental disorders were more likely to be engaged in risky behaviors during pregnancy. Women in the study group were at a 4-fold and a 2-fold greater risk of smoking and attending fewer ANC visits, respectively, than their healthy counterparts.

Maternal smoking is significantly associated with smaller birth weight and length, as well as it causes poor birth outcomes [16–18]. The higher rates of prematurity, stillbirths, major malformations, lower Apgar scores and newborns with low birth weight (LBW) were observed more often in the study group, but the difference was not significant, most probably because the sample size was not large enough. However, the results suggest a possible association between mental disorders and a negative impact on newborn health. The same association was noticed in other studies [19–22]. Although we did not find any significant difference in the rate of delivering premature newborns, there are contradictory findings on whether mental disorders and being exposed to psychotropic medication are risk factors for having a premature newborn [9, 23].

We found a significant difference in the rates of delivering SGA newborns between the groups: a pre-existing mental disorder doubles the risk of giving birth to an SGA newborn. These findings are consistent with other studies on the effects of mental disorders on pregnancy [24, 25].

Women with pre-existing mental disorders had a longer postpartum recovery time in hospital. According to the official statistical data, an average hospital stay in the psychiatric and obstetric departments of Kaunas Klinikos is 13.5 and 4.07 days, respectively [26]. There were women transferred from the psychiatric department to the obstetric department because they were in labor and then after giving birth transferred back to the psychiatric department to continue their treatment. Some were transferred to the psychiatric department after giving birth for treatment because they had relapsed. Women from the study group who were transferred to or from the psychiatric department had an increased average length of hospital stay up to 32 days.

The study was conducted in the University Hospital of tertiary care, which is the largest healthcare institution in Lithuania. The University Hospital has been recognized as a Baby Friendly Hospital because of how it protects, promotes, and supports breastfeeding, and for its facilities providing maternity and newborn services. However, women with pre-existing mental disorders were less likely to breastfeed their newborns: the relative risk of not breastfeeding increased by nearly 7 times.

There was no significant difference in the method of delivery between the study group and healthy controls. Both elective and emergency Caesarean sections (CSs) were performed in 20.6% and 23.2% of the women from the study and control groups, respectively. However, a matched, controlled cohort study by Howard et al. [15] reported a significant difference between the groups (20% vs. 14%) with the study group requiring CSs more frequently. This can be explained, however, by differences in methodology and by the different clinical practices used in different countries and hospitals. Our study was performed in a highly specialized, tertiary hospital with a considerable number of high-risk deliveries, while the previously mentioned study used records from a General Practice Research database [15]. The results of another study, which was performed in two tertiary obstetric hospitals, are closer to those found by us, with 20.2% of women with mental disorders requiring elective and 16.1% having emergency CSs [27].

### Limitations

Because of the infrastructure of the health system and data protection restrictions in Lithuania, it was not possible to access patient records from the participants’ primary care physicians or data from their psychiatrists. Only information available during delivery, in birth registry records, was used for the identification of cases; therefore, a significant number of pregnant women with mild mental disorders may have gone unmentioned during birth and therefore been missed. There may be some inaccuracies in the medical records regarding whether patients were receiving psychotropic medication at the time of their pregnancy. The stigma of mental illness must also be taken into account, since some of the patients from the control group may have concealed that they also had a mental disorder. The motivation for doing so is reinforced by fear of custody loss after becoming a parent [28].

Since the study was performed in the Department of Obstetrics and Gynecology, Lithuanian University of Health Sciences Kauno Klinikos – a highly specialized hospital – it may not represent the most common problems that medical professionals face in their daily work, with regards to pregnant women with mental disorders. For example, pregnant women from the control group may have had higher incidence rates of concomitant diseases than women who give birth in less specialized hospitals, and this could have altered the results of this study.

Further research in this field is essential, and a prospective design would be more informative. A prospective study would enable researchers to perform more homogenic and objective analysis of patients with mental disorders, to access more data on their psychotropic drug use during pregnancy, and to access data concerning long-term outcomes for both newborns and mothers.

## Conclusions

To conclude, pregnant women with mental disorders were more likely to be less educated, unemployed, single, smokers and to have concomitant diseases. Moreover, they did not know the date of their last menstrual period, did not seek ANC more often, had fewer attendance visits, gained less than 10 kg of weight per pregnancy, and had more hospital admissions. As compared with healthy controls, mentally ill pregnant women delivered small-for-gestational-age newborns significantly more often and were less likely to breastfeed. Other neonatal outcomes tended to be worse, but the difference was not significant. No difference in the incidence of pregnancy-related diseases or the delivery methods chosen was found when comparing the groups.

## Data Availability

As the data were acquired in Lithuanian, the data sets analyzed for this report will be made available from the corresponding author K. S. upon reasonable request.

## Abbreviations

SGA: Small for gestational age
ICD – 10: International classification of diseases – 10
UTI: Urinary tract infection
CS: Caesarean section
LMP: Last menstrual period
ANC: Antenatal care
GDM: Gestational diabetes mellitus
LBW: Low birth weight
